# Inter-Individual Variability in Motor Output Is Driven by Recruitment Gain in the Corticospinal Tract Rather Than Motor Threshold

**DOI:** 10.3390/brainsci12101401

**Published:** 2022-10-17

**Authors:** Arkaprovo Sarkar, Alish Dipani, Giorgio Leodori, Traian Popa, Panagiotis Kassavetis, Mark Hallett, Nivethida Thirugnanasambandam

**Affiliations:** 1 Human Motor Neurophysiology and Neuromodulation Lab, Department of Biosciences and Bioengineering, Indian Institute of Technology Bombay, Mumbai 400076, India; 2 National Brain Research Centre (NBRC), Manesar 122052, India; 3 Department of Human Neuroscience, Sapienza University of Rome, 00185 Roma, Italy; 4 Neuromed Mediterranean Neurological Institute, Scientific Institute for Research, Hospitalisation and Healthcare (I.R.C.C.S.), 86077 Pozzilli, Italy; 5 Human Motor Control Section, National Institute of Neurological Disorders and Stroke, Bethesda, MD 20892, USA; 6 Department of Life Sciences, Swiss Federal Institute of Technology (EPFL), 1950 Sion, Switzerland; 7 Department of Neurology, University of Utah, Salt Lake City, UT 84112, USA

**Keywords:** input output curve, transcranial magnetic stimulation, motor threshold, inter-individual variability, peak slope

## Abstract

Variability in the response of individuals to various non-invasive brain stimulation protocols is a major problem that limits their potential for clinical applications. Baseline motor-evoked potential (MEP) amplitude is the key predictor of an individual’s response to transcranial magnetic stimulation protocols. However, the factors that predict MEP amplitude and its variability remain unclear. In this study, we aimed to identify the input–output curve (IOC) parameters that best predict MEP amplitude and its variability. We analysed IOC data from 75 subjects and built a general linear model (GLM) using the IOC parameters as regressors and MEP amplitude at 120% resting motor threshold (RMT) as the response variable. We bootstrapped the data to estimate variability of IOC parameters and included them in a GLM to identify the significant predictors of MEP amplitude variability. Peak slope, motor threshold, and maximum MEP amplitude of the IOC were significant predictors of MEP amplitude at 120% RMT and its variability was primarily driven by the variability of peak slope and maximum MEP amplitude. Recruitment gain and maximum corticospinal excitability are the key predictors of MEP amplitude and its variability. Inter-individual variability in motor output may be reduced by achieving a uniform IOC slope.

## 1. Introduction

**Figure 1:**
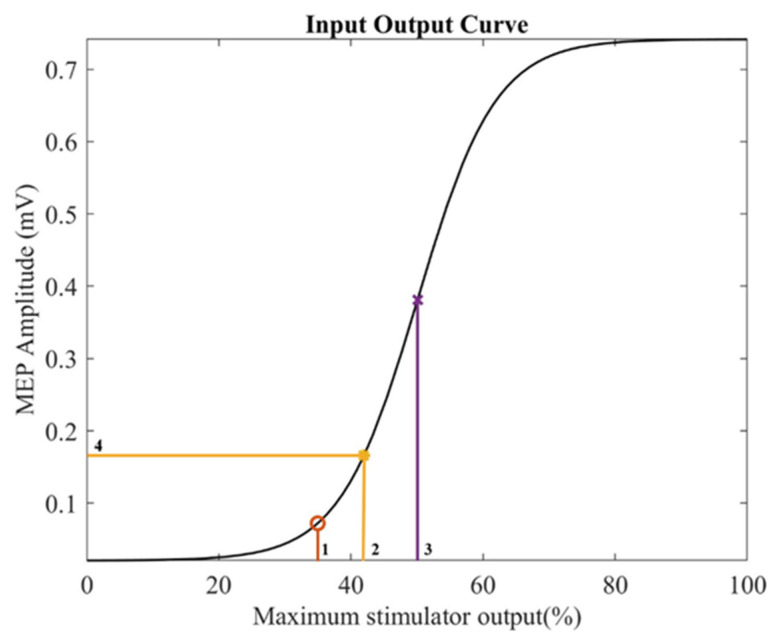
Input–output curve of one participant from this study. The labels describe the following parameters: (1) Resting motor threshold (RMT), (2) 120% RMT, (3) Intensity at half-maximum amplitude (S50) (4) MEP amplitude at 120% RMT (MEP_amp_). The offset and MEP_max_ is visible in the IOC as maximum and minimum MEP values, respectively. Peak Slope (PS) is the maximum slope of the curve which can be obtained at S50.

Transcranial magnetic stimulation (TMS) is a non-invasive brain stimulation technique that works on the principle of Faraday’s law of electromagnetic induction. A rapidly changing electric field in the coil generates a magnetic field that penetrates the skull painlessly and induces electrical current over the cortical area of interest. If strong enough, the induced electric current can trigger an action potential in the neurons of the outermost layers of the brain [1]. If the coil is positioned over the motor cortex (M1), the induced current activates the pyramidal neurons of the corticospinal tract trans-synaptically, triggering volleys down the corticospinal pathway resulting in the generation of motor-evoked potentials (MEPs) in target muscles [2]. The amplitude of these MEPs recorded using surface electromyography (EMG) is a good measure of corticospinal excitability (CSE) [3,4]. Measuring MEPs at a range of stimulation intensities rather than at a single intensity could offer valuable insights to the physiological mechanisms underlying motor excitability [5,6,7,8]. Plotting the MEP amplitudes against the corresponding stimulation intensities yields an MEP recruitment curve or the input–output curve (IOC) (Figure 1), which can be described by the Boltzmann sigmoid function equation [9,10,11,12]. Different aspects of the IOC represent different physiological characteristics of motor excitability.

The minimum stimulation intensity needed to generate a recordable EMG response agreed to be around 50 µV peak-to-peak from target muscle at rest in at least 50% of the trials is often defined as resting motor threshold (RMT) [13]. It corresponds to the stimulation intensity at the first positive inflection point on the IOC. The RMT depends on the membrane potential of the neurons stimulated by TMS, excitability of synapses between excitatory inputs and corticospinal neurons at the cortex level, and the excitability of synapses between corticospinal terminals and neurons at the spinal cord level. These factors contribute to the generation of MEP at the threshold intensity [14]. Stimulation intensity beyond the motor threshold generates the exponential phase of the IOC until it reaches a plateau representing maximum MEP output (MEP_max_) achievable in the target muscle. The saturation point in MEP amplitude indicates recruitment of all corticospinal neurons available for TMS stimulation. MEP_max_ is influenced by the number of corticospinal volleys generated by the TMS pulse, the number of facilitatory synapses per corticospinal fibre and the total number of available corticospinal fibres [14]. All subjects may not necessarily show MEP max even at 100% stimulator output intensity, which represents an inherent technical limitation. The slope of the IOC is highest at S50 (stimulation intensity that elicits a response equal to 50% of the maximum) and this peak slope (PS) represents the distribution of excitability of synapses in the cortex and spinal cord. PS of the sigmoid curve represents the recruitment gain and the MEP amplitudes represent the output of the descending CS pathway [9,15,16,17,18]: the activation of large number of corticospinal fibres within a narrow stimulation intensity range results in a steeper IOC. This is the reason why an activated muscle, which is driven by more uniform corticospinal synaptic excitability, will have a steeper IOC and higher PS than the same muscle at rest [19].

A suprathreshold TMS pulse usually activates both excitatory and inhibitory neurons within the primary motor cortex, therefore representing the net activity of the corticospinal pathway [4]. By adjusting the TMS pulse intensity and waveform, it is possible to activate different classes of neurons, enabling the study of corticospinal excitability in much finer detail [20,21]. However, the major challenge with TMS studies is the large variability in MEP amplitude both within and across individuals. Carrol et al. (2001) and Kukke et al. (2014) [10,19] have addressed the problem of MEP amplitude variability within individuals by suggesting measures that increase reproducibility. Kukke et al. (2014) demonstrated excellent test–retest intra-individual reliability for all the parameters of the IOC when a minimum of 40 TMS pulses (2 pulses/intensity and 20 intensities distributed equally between 5% and 100% MSO) were used to construct the IOC. However, the issue of large inter-individual variability [22] in MEP amplitude across individuals remains unaddressed. Many studies have attempted to identify the factors driving this variability. Factors such as age [23,24], gender [23,25], stimulation intensity [26,27] and the stimulated hemisphere [28,29] seem to be important contributors to the inter-individual variability. However, the findings have not been consistent across studies, most likely due to small sample sizes [30]. Recent work by Corp et al. collated data from 35 studies and revealed that the response of healthy individuals to repetitive and to paired-pulse TMS protocols is best predicted by the baseline MEP amplitude [31,32]. Leodori et al. showed that the variability in plasticity associated with theta burst stimulation depends partially on baseline corticospinal excitability [33]. Most TMS studies use the MEP amplitude at stimulation intensity equal to 120% RMT (MEP_amp_) to measure baseline corticospinal excitability [34,35]. Some others use 1 mV intensity. Although the stimulation intensity could be optimized using 120% RMT intensity across individuals, the variability in their response to TMS protocols still persists.

Corp et al. (2021) [32] rightly state that the non-availability of IOC data is an important limitation of their study. This is because IOC data contain information about corticospinal output across the entire spectrum of stimulation intensities, serving as an accurate representation of motor excitability. Unfortunately, the majority of the studies do not record IOC, primarily due to time constraints. In the current study, we aimed to identify the most important IOC parameters that can predict the MEP amplitude at stimulation intensity equal to 120% RMT. For this, we collated IOC data recorded from individuals who participated in three different studies at the Human Motor Control Section, NINDS, Bethesda, USA. This is the first study that has examined the role of IOC parameters in predicting the motor output using a relatively large sample size. We also intended to identify IOC parameters that drive the inter-individual variability of MEP_amp_. Since the IOC offers a more detailed characterisation of CSE, we expect to obtain valuable insights into the physiological processes that drive inter-individual variability in motor output, a problem that limits the clinical potential of non-invasive brain stimulation.

## 2. Materials and Methods

### 2.1. Participants

The IOC data from 84 healthy adult individuals (mean age: 38 ± 12 years; 39 females) who participated in three different TMS studies (15, 33 & 36) at the Human Motor Control Section, NINDS, Bethesda, USA were analysed. All three studies were approved by the Combined Neuro Sciences IRB of the National Institute of Neurological Disorders and Stroke (NINDS) and conformed to the guidelines of the Declaration of Helsinki. All participants gave written informed consent before participation. None of the participants had any contraindications for undergoing TMS and no adverse events were reported. We excluded data from 6 subjects because their IOC did not saturate at 100% stimulator intensity. We also excluded 3 subjects whose IOC parameters were beyond 3 standard deviations (or 99.73% confidence interval) from mean value. The data from the remaining 75 subjects were included for analysis.

### 2.2. Input–Output Curve (IOC)

Participants were seated comfortably on a reclining chair with their right hand resting on a pillow by the side. Surface EMG electrodes were placed over one of the hand muscles–abductor pollicis brevis (APB) or abductor digiti minimi (ADM). Single monophasic TMS pulses were delivered using Magstim 200^2^ (Magstim Ltd., Whitland, UK) connected to a 70 mm figure-of-eight coil. The coil was positioned at an angle of about 45° from the midline and the handle pointing backwards so as to deliver a postero-anteriorly directed current in the brain. The motor hotspot of the hand muscle was first identified while the hand was fully relaxed. The EMG signal was amplified (gain = 1000) and filtered (20–2000 Hz) by an EMG system (Nihon Kohden Neuropack MEB 2200, v.08.15, Tokyo, Japan). The signal was then digitized at 5000 Hz using the CED micro 1401 laboratory interface (Cambridge Electronic Design Ltd., Cambridge, UK) and stored for offline analysis using their Signal version 7 software. To record the IOC, TMS pulses were delivered at 20 different stimulator intensities—from 5% to 100% of maximum stimulator output in increments of 5%. The order of the stimulation intensities was randomized. Thus, 60 pulses with at least 3 pulses per intensity were delivered. We estimated the peak-to-peak MEP amplitude for each trial, plotted it against the corresponding stimulation intensity, and fitted a Boltzmann sigmoid function to the data [9].

Boltzmann Sigmoid Function:

*MEP_x_* = *Offset* + (*MEP_max_* − *Offset*)/[1 + exp{(*S50* − *x*)/*k*}]

The variables are:

*MEP_x_* = MEP amplitude at *x* % maximum stimulator output.

*Offset* = The offset value or minimum MEP amplitude obtained for the subject.

*MEP_max_* = The MEP amplitude where saturation point of the curve has been reached.

*S50* = Stimulator intensity that elicits half the maximum MEP amplitude.

*k* = Slope of the curve.

Additionally, for each subject, we estimated parameters such as Motor Threshold (MT), Peak Slope at the midpoint of the curve (PS), MEP amplitude at 120% RMT (MEP_amp_), and the S50 from the IOC. MT was obtained by calculating the mean of the intensity corresponding to the point of maximum curvature at the rising phase of the IOC and the intensity that elicited 5% of maximum MEP amplitude. All the above analyses were performed using custom MATLAB R2022a (The MathWorks, Inc., Natick, MA, USA) scripts.

Since IOC was obtained from abductor digiti minimi (ADM) in some subjects and abductor pollicis brevis (APB) in others, we compared the IOC parameters between the two groups to ensure that there was no significant difference before pooling them together (Supplementary Table S1).

### 2.3. Predicting MEP Amplitude

The parameters—PS, MEP_amp_ and MEP_max_—were not normally distributed in the dataset as tested by Shapiro–Wilk test (Supplementary Table S2). Hence, all IOC parameters were log-transformed to achieve normal distribution and Pearson’s correlation was performed. A principal component analysis (PCA) correlation circle was also generated to better visualize the results [36]. The PCA correlation circle was generated using FactoMineR package for multivariate analysis in R [37]. To examine the inter-individual variability in MEP amplitude at 120% RMT, a General Linear Model (GLM) was built by taking the other parameters (PS and MEP_max_) as independent variables. Z-score normalization was performed on the independent variables before running the GLM. Non-significant predictors were removed and the GLM was run again to derive an accurate estimate of the contribution of the significant predictors. The variance inflation factor (VIF) was also checked in the final model to ensure that there was no multicollinearity [38].

Furthermore, to evaluate the performance of the GLM in predicting MEP amplitude at 120% intensity, we performed 5-fold cross validation resampling procedure where the dataset was divided into 5 groups with equal number of samples. The predictive model is trained on 4 such groups or “folds” and its accuracy at prediction was tested on the remaining fifth group by checking root-mean-square error (RMSE). This was repeated until all 5 folds were tested against. If a predictor variable had a strong non-linear relationship with MEP_amp_, a GLM model would not account for it. Hence, we compared the accuracy of our GLM with a non-linear model such as the Random Forest (RF) machine learning algorithm using a similar 5-fold cross validation procedure. The evaluation of the performance of the models was performed using a custom script in R Studio using ‘train’ function available in the ‘caret’ package (version 4.47) [39]. The RF used 500 decision trees with 4 variables at each split as optimized by the function according to performance. To ensure that there is no seeding bias, we ran the above analysis 1000 times and then compared the mean and standard deviation of RMSE of the two models. We used Student’s two-sample *t*-test when samples had equal variance and Welch’s two-sample *t*-test for samples with unequal variance as determined by an F test. All of the analyses mentioned above were performed using custom scripts in R.

### 2.4. Variability in MEP Amplitude

We used coefficient of variation (CV) as a measure of inter-individual variability [40], which was calculated using the following formula:

CV = SD/mean

where CV = Coefficient of variation; SD = Standard deviation; mean = Mean value of parameter.

**Figure 2:**
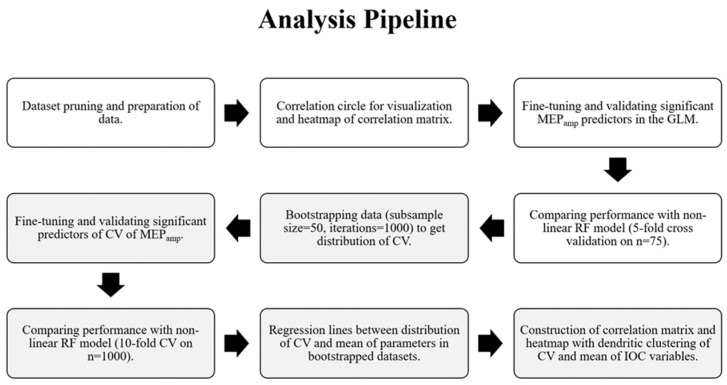
Flowchart of data analysis pipeline. (MEP_amp_ = MEP amplitude at 120% resting motor threshold, GLM = General Linear Model, CV = Coefficient of Variation, RF = Random Forest, IOC = Input–Output Curve).

To assess the variability of the different IOC parameters, the data were bootstrapped without replacement by choosing subsamples of 50 subjects from a total sample size of 75 in 1000 iterations. This yielded 1000 CV values of MEP_amp_, MT, PS, MEP_max_ and S50 that represented the distribution of inter-individual variability within our dataset. The bootstrapped CV values for each predictor variable were plotted against the CV values of MEP_amp_. To identify the IOC parameters that best predicted the variability in MEP_amp_, a GLM was built using the CVs of the bootstrapped variables. Variables that showed high collinearity (VIF ≥ 2.5) [38,41,42] and those with *p* value >0.05 were excluded and the GLM was re-run with the significant, non-collinear variables. The GLM and a non-linear RF model were trained on the bootstrapped data using 10-fold cross validation, as described previously. The RF model used 500 decision trees with 2 variables at each split as optimized according to performance. To avoid seeding bias, the calculated mean RMSE from 1000 iterations from both the models were calculated and compared. A flowchart illustrating the entire data analysis pipeline described above is shown in Figure 2.

## 3. Results

### 3.1. Correlation Analysis

**Figure 3:**
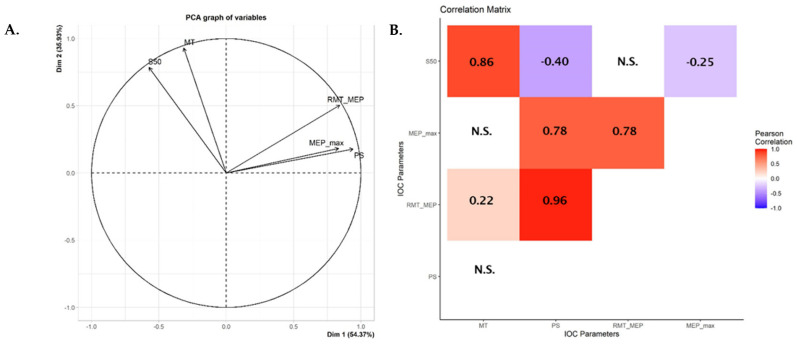
Correlation between the different IOC parameters. (**A**) PCA correlation circle where the *x* and *y* axis represent the first two principal components obtained from PCA. Parameters with arrows in same direction are strongly correlated while orthogonal parameters are not correlated. (**B**) A Pearson’s correlation matrix for the significant parameters is shown. (N.S. = non-significant correlation, *p* > 0.05).

A PCA correlation circle and a correlation matrix were generated using the MT, PS, MEP_amp_, MEP_max_ and S50 parameters obtained from the IOC (Figure 3). Among them, the following pairs of IOC parameters showed significant levels of correlation: MT vs. S50 (rho = 0.86, *p* < 0.0001), PS vs. 120% MEP_amp_ (rho = 0.96, *p* < 0.0001), PS vs. MEP max (rho = 0.78, *p* < 0.0001), PS vs. S50 (rho = −0.40, *p* = 0.0004), MEP_amp_ vs. MEP_max_ (rho = 0.78, *p* < 0.0001) and MEP_max_ and S50 (rho = −0.25, *p* = 0.0325). The correlations between MT vs. PS (rho = −0.01, *p* = 0.9071), MT vs. MEP_amp_ (rho = 0.22, *p* = 0.0547), MT vs. MEP_max_ (rho = −0.15, *p* = 0.2051) and MEP_amp_ vs. S50 (rho = −0.15, *p* = 0.1954) were not significant. The regression curves for each pair of variables and their Pearson’s correlation coefficients are discussed in the Supplementary Document (Supplementary Figure S1).

### 3.2. General Linear Model (GLM) for Predicting MEP Amplitude

A GLM was built using MEP_amp_ as dependent variable and z-score normalized MT, PS, MEP_max_ and S50 as predictor variables (Supplementary Table S3). Among the predictor variables, the VIFs of S50 and MT were 10.814 and 8.865 indicating very high collinearity, making the coefficients non-interpretable in the GLM. Hence, another GLM was generated after removing S50 which had the highest VIF. The model had the following properties: residual SE = 0.2301 on 71 degrees of freedom, r^2^ = 0.9468, adjusted r^2^ = 0.9446, F(3,71) = 421.4, *p* < 0.0001 (Table 1).

The final model was a very strong predictor of MEP_amp_ (adjusted R-squared = 0.9446), indicating that MT, PS and MEP_max_ could predict more than 94% of the variance in MEP_amp_. Since all the predictor variables were z-score normalized before running the model, a larger coefficient indicates greater contribution of that predictor in predicting MEP_amp_. The coefficient of PS was much larger than that of MT or MEP_max_. Thus, in our model, PS is the crucial parameter that best predicts MEP_amp_. VIF < 2.5 for all predictors indicating the absence of significant collinearity; hence, the coefficients accurately reflect the contribution of the predictors. The accuracy of the GLM did not change significantly when S50 was removed as a predictor (Supplementary Figure S2), implying that it was not as important in predicting MEP_amp_ as the other variables.

### 3.3. Strength of the Predictive Model

**Figure 4:**
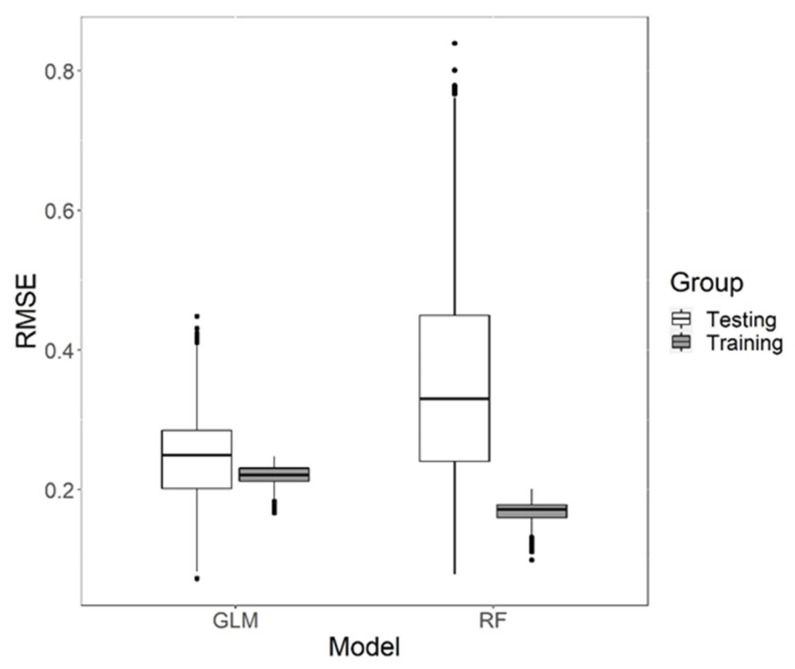
Performance of the General Linear Model (GLM) and Random Forest (RF) model after 5-fold cross validation iterated 1000 times to eliminate seed bias for the training and testing data. The solid line represents median Root Mean Square Error (RMSE), the upper and lower hinges form the 1st and 3rd quartiles while the whiskers extend to the highest and lowest value within 1.5 times inter-quartile range from the two hinges. The black dots represent outliers beyond the range of whiskers.

The root-mean-square error (RMSE) is a measure of the accuracy of continuous predictive models. The mean RMSE obtained through five-fold cross validation across 1000 iterations of the GLM was compared with the RMSE of a random forest (RF) machine learning model which is a robust non-linear predictive model (Figure 4). The significant variables for the GLM were MT, PS and MEP_max_ which showed a linear relationship with MEP_amp_. In the non-linear RF model, MT, PS, MEP_max_ and S50 were taken into account as predictor variables for MEP_amp_. The testing RMSE results of both models showed a significant difference in variance (F(4999, 4999) = 0.1899, *p* < 0.0001); hence, we used Welch’s two-sample *t*-test and found a significant difference between the means (t(6831.4) = −50.736, *p*< 0.0001).

The overall predicting power of the GLM (RMSE (mean ± SD) = 0.2453 ± 0.0583) was significantly better than that of the RF model (RMSE = 0.3499 ± 0.1338). This implies that a simple linear model with MT, PS and MEP_max_ as predictors is more accurate than a RF model which takes into account non-linear relationships between all five IOC variables.

We also checked for overfitting in both models by comparing their training versus testing performance (Supplementary Table S4). Although both models showed significant difference between training and testing RMSE, the RF model had a much larger difference than GLM.

### 3.4. Analysing the Variability of Parameters

Having found that MT, PS, and MEP_max_ are significant linear predictors of MEP_amp_, we wanted to see how the variability of these predictors affected the variability of MEP_amp_. To measure the variability within the entire dataset, the coefficients of variation (CV) of all the IOC parameters were calculated (Supplementary Figure S3). The CV of PS, MEP_amp_ and MEP_max_ were much larger than that of MT and S50 implying larger inter-individual variability observed in those three parameters.

Using bootstrapping without replacement, we obtained a distribution of CV values of all the IOC parameters within our dataset. We wanted to see whether this distribution of CV of the predictor variables could be used to predict the CV of MEP_amp_, thereby directly associating the inter-individual variability of IOC parameters to that of MEP amplitude. For this, we built a GLM using CV of all the previously used predictor variables to predict the CV of MEP_amp_. We then determined the GLM parameters from 10-fold cross validation model training using bootstrapped data (Supplementary Table S5). Our results showed that CV of S50 and MT show collinearity; hence, we removed the predictor with the largest VIF (S50, VIF = 3.6727) and ran the model again (Supplementary Table S6). After removing the CV of S50 from the model, the CV of MT became a non-significant predictor (*p* = 0.192). Hence, we construct a final model with only the CV of PS and CV of MEP max as the predictors. The model had the following properties: residual SE = 0.0259 on 997 degrees of freedom, r^2^ = 0.6425, adjusted r^2^ = 0.6418, F(2,997) = 895.9, *p* < 0.0001 (Table 2).

The final GLM could predict about 64% of the change in CV of MEP_amp_ using the variability of only PS and MEP_max_. The coefficients of the CV of MEP_max_ (β = 0.7482) are much higher than that of PS (β = 0.4781) in the final model indicating that it is a more important predictor of MEP_amp_ variability. A GLM using the CV of all the IOC parameters as predictors has similar performance to a GLM using only PS and MEP_max_, thus validating that only CV of PS and CV of MEP_max_ are the crucial parameters in our linear model (Supplementary Figure S4). There is also a strong correlation between CV of MEP_amp_ with CV of PS (rho = 0.66, *p* < 0.0001) and CV of MEP_max_ (rho = 0.64, *p* < 0.0001) (Supplementary Figure S5).

**Figure 5:**
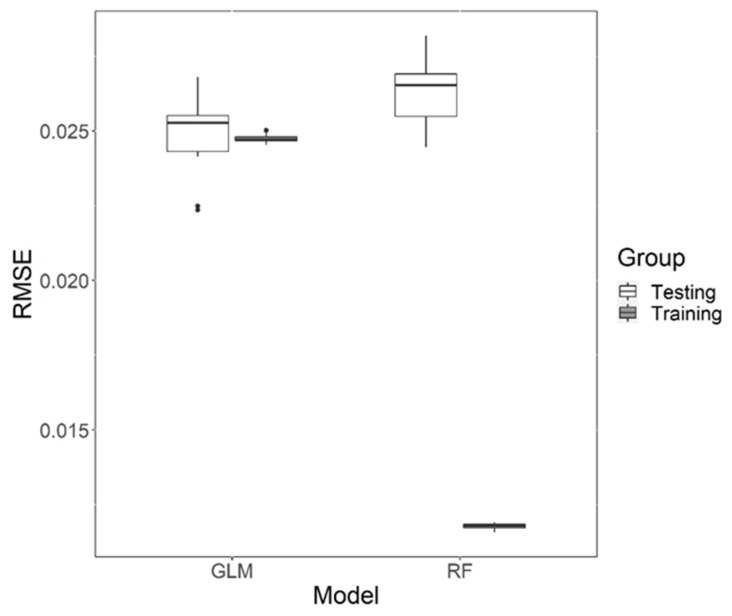
Performance of the General Linear Model (GLM) and Random Forest (RF) model after 10-fold cross validation in 1000 iterations. The solid line represents median Root mean square error (RMSE) while the whiskers represent maximum and minimum value within 1.5 times of inter-quartile range from the hinges. The black dots represent outliers beyond the range of whiskers. (GLM: Testing = 0.0248 ± 0.0018; Training = 0.0247 ± 0.0002; RF model: Testing = 0.0262 ± 0.0019, Training = 0.0118 ± 0.0001).

We checked the performance of the GLM with only these two predictors against a RF algorithm using 10-fold cross validation run through 1000 iterations to minimize bias (Figure 5). The testing RMSE results of both models had unequal variances (F(9999, 9999) = 80.149, *p* < 0.0001); hence, we used Welch’s two-sample t-test which showed a significant difference between the testing RMSE means of the two models (t(10248) = 3.0692, *p* = 0.0021) with the GLM model having better performance than the RF model.

We found significant difference between training and testing RMSE in both models (Supplementary Table S7). However, the testing versus training RMSE of the RF model had a large difference, indicating overfitting to the training dataset.

## 4. Discussion

This is the first study to examine the role of different IOC parameters on MEP amplitude in a large sample size. Our results show that MEP_max_ and peak slope of IOC are the key predictors of MEP amplitude and its variability. We also show that MT and S50 do not contribute significantly towards the inter-individual variability in motor output.

Kemlin et al. (2019) [36] conducted a study on the IOC parameters of 24 healthy subjects and 40 stroke survivors. In healthy subjects, they found strong positive correlation of PS with MEP amplitude at 140% RMT (rho = 0.76, *p* < 0.001), PS and MEP_max_ (rho = 0.85, *p* < 0.001) and between MEP_max_ and MEP amplitude at 140% RMT (rho = 0.73, *p* <0.001). Our study used MEP amplitude at 120% RMT instead of 140%, but our results are similar. They reported non-significant correlation between 140% RMT MEP and S50, similar to our 120% RMT and S50. However, we found significant negative correlation between PS and S50 and between MEP_max_ and S50, while they did not find any significant correlation. A point of note is that they used the measure of %RMT on the *x*-axis for constructing their IOC; thus, their IOC slope shows recruitment gain for different increments of stimulator output intensity. In our study, we used absolute maximum stimulator output percentage as the *x*-axis measure. This might be the reason for the difference in some of the results. In addition to just examining correlations, we also delve deeper into identifying the crucial parameters that drive motor output and its variability.

After removing non-significant and high collinearity variables, our GLM revealed MT, PS, and MEP_max_ as significant predictors of MEP_amp_. Since all the predictors were z-score normalized, their coefficients indicate their level of importance in the linear model. PS showed the highest positive correlation with MEP_amp_ and was its best predictor. Our simple linear model with only the MT, PS, and MEP_max_ as predictors was sufficient to predict MEP_amp_ with good accuracy implying a strong linear relationship between these IOC parameters and MEP amplitude. MT is determined significantly by the synaptic excitabilities of the spinal and cortical neurons that constitute the corticospinal pathway, while PS represents the distribution of this excitability [14]. MEP_max_ reflects the excitability ceiling at a particular brain state. These parameters combined represent the excitability of the subset of corticospinal fibres activated by the TMS pulse at a certain brain state; therefore, it is not surprising that they are sufficient to accurately predict the MEP at a particular stimulator intensity.

Among the IOC parameters, the strongest correlation was observed between PS and MEP_amp_. This is expected because a steeper slope in the rising phase of the IOC corresponds to higher recruitment gain in the corticospinal pathway resulting in larger MEP at 120% RMT intensity. S50 showed moderate and weak negative correlation with PS and MEP max, respectively. It is worth noting that we did not find any significant correlation between PS and MT, in contrast to the findings of Peterchev et al. (2013) [43], which showed significant negative correlation between them. This might be due to methodological differences as we used the standard Magstim 200^2^ monophasic pulse of 80 μs pulse width, while they used controllable pulse width TMS with monophasic pulses of 30 μs, 60 μs and 120 μs pulse widths. Moreover, they studied a much smaller number of subjects (n = 12), which might be the main reason for this difference.

**Figure 6:**
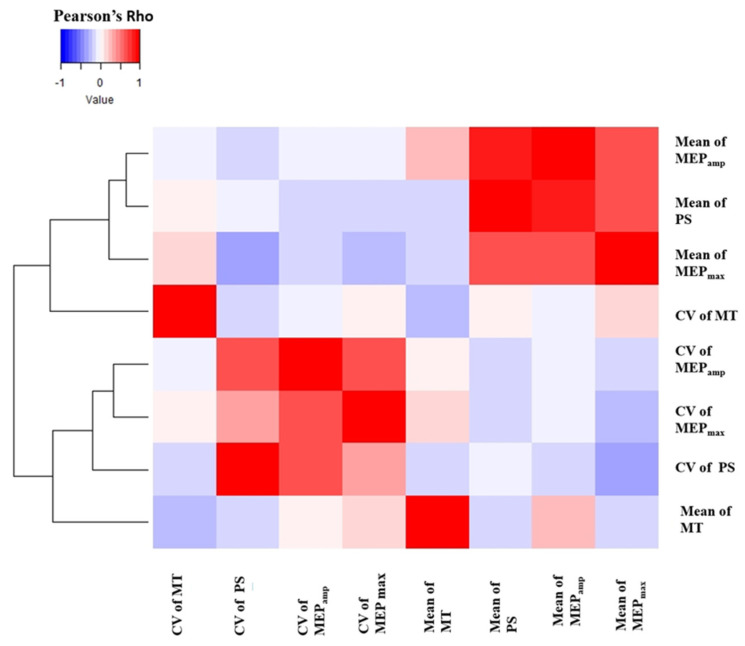
Correlation heatmap and dendrogram showing the relationships between the Coefficient of Variation (CV) and the means of all IOC parameters. PS—peak slope; MEP_amp_—motor evoked potential amplitude at 120% resting motor threshold; MT—motor threshold; MEP_max_—maximum MEP amplitude.

One of our primary objectives was to find a correlation between inter-individual variability of IOC parameters with that of MEP_amp_. We used the CV of the different IOC parameters as a measure of inter-individual variability. PS, MEP_amp_ and MEP_max_ had the highest CV values in our dataset. Furthermore, we showed that variability in the MEP_amp_ across individuals is mainly driven by the PS and MEP_max_, which reflect the recruitment gain and the maximum excitability of the motor neuronal pool at a certain brain state, respectively. However, our GLM could explain only about 64% of variability in MEP_amp_ implying that the inter-individual variability in MEP amplitude can only be partially described by the inter-individual variability in IOC parameters. A recent study by Goetz et al., 2022 has revealed the crucial role of spinal excitability component in determining the variability in motor output. The relationships between different IOC parameters and their variabilities are summarized by Figure 6.

Our study shows that inter-individual variability of MEP amplitude can be accounted for to a fair extent by the inter-individual variability of PS and MEP_max_. MEP_max_ in an individual may be constant at a particular brain state, whereas the recruitment gain (PS) may be modulated by changing the stimulation parameters, for example, the TMS pulse width as reported previously by Peterchev et al. (2013) [43]. They demonstrated that pulse width of monophasic single pulse TMS has a positive correlation with the slope of the IOC. This might be because the increased energy transferred by a larger pulse width might recruit a larger number of target cortical neurons or it might also employ a different corticospinal excitatory network. Both may result in a higher recruitment gain and thereby a steeper PS of the IOC. The implication would be that we can obtain IOC with uniform PS across individuals by modulating the width of the stimulating pulse. Furthermore, we speculate that individualizing the TMS pulse widths to achieve similar recruitment gains across subjects would reduce the inter-individual variability of MEP amplitude. Future studies should test this hypothesis which is possible using a controllable TMS system [44,45,46]. One of the main limitations of our study is that we estimated MEP amplitude at 120% RMT from the Boltzmann equation rather than measuring it experimentally. This is because we performed a retrospective analysis on data collected in past studies in our lab. Our prediction model needs to be tested on experimental data. That said, this is still an important study where parameters from such a large number of complete IOCs (n = 75) across multiple studies have been analysed to determine their relationships with MEP amplitude. We would also like to point out that our study has focused entirely on the output of the motor cortex to TMS while the muscle was at rest; therefore, MEP amplitude was used as the sole marker of corticospinal excitability. This is because MEP amplitude is the most widely recorded outcome measure in TMS studies for which we have a large amount of data from multicentric studies [31,32]. However, recent studies have revealed the importance of cortical silent period as a neurophysiological index to assess the status of cortical and spinal motor neurons [47]. Considering other such parameters may further enhance our understanding of the neural underpinnings of inter-individual variability. Furthermore, the motor cortex has been the preferred brain region for TMS studies due to its superficial location and availability of an objective peripheral outcome measure—MEP amplitude. Although the results of the current study are extremely useful in understanding the neurophysiological mechanisms underlying TMS, their implications for other brain regions need to be considered with caution owing to differences in their neuronal architecture and physiological properties. A well-known example is the significant difference in the resting motor threshold of the primary motor cortex and the phosphene threshold of the primary visual cortex [48]. Hence, we cannot assume that the IOC properties of the primary visual cortex would be similar to that described in our study. Another important aspect that the current study could not address is the influence of cortico-cortical interaction [49] and instantaneous brainstate [50] on motor output and their temporal dynamics [51]. These are more complex, yet extremely important questions that remain to be addressed in future studies.

## 5. Conclusions

In summary, we have shown that PS, RMT and MEP_max_ can predict the MEP amplitude at 120% RMT. MEP_max_ and PS are the most important linear predictors of inter-individual variability in MEP amplitude at 120% RMT intensity. RMT, which is commonly used as a reference to individualize TMS stimulation, is not a good predictor of MEP amplitude at 120% RMT, and is not significant in predicting its variability across subjects.

## Figures and Tables

**Table 1:** **General Linear Model** (GLM) of MEP_amp_ with MT, PS and MEP_max_ as predictor variables.

	Coefficient (β)	Standard Error (SE)	t-Stat	*p*-Value	VIF
Intercept	1.3733	0.0266	51.697	<0.0001	
MT	0.3210	0.0271	11.856	<0.0001	1.0252
PS	0.7650	0.0365	20.928	<0.0001	1.8684
MEP_max_	0.2270	0.0368	6.166	<0.0001	1.8958

**Table 2:** GLM for predicting CV of MEP_amp_ with CV of PS and MEP_max_ as predictor variables.

	Coefficient (β)	Standard Error (SE)	t-Stat	*p*-Value	VIF
Intercept	−0.0235	0.0175	−1.348	0.178	
CV of PS	0.4781	0.0187	25.581	<0.0001	1.1138
CV of MEP_max_	0.7482	0.0315	23.780	<0.0001	1.1138

## Supplementary Materials

The following supporting information can be downloaded at: https://www.mdpi.com/article/10.3390/brainsci12101401/s1, Figure S1: Correlation matrix with regression line; Figure S2: Performance of two different GLMs predicting MEP_amp_; Figure S3: Coefficients of variation (CV) for the IOC parameters; Figure S4: Performance of two different GLMs predicting CV of MEP_amp_; Figure S5: Regression lines between the final two predictors of MEP_amp_ CV; Figure S6: Regression lines between all IOC parameters along with their Pearson’s correlation coefficients. Table S1: Result of Welch’s *t*-test; Table S2: Results of the Shapiro-Wilk test; Table S3: GLM of MEP_amp_ with MT, PS, MEP_max_ and S50 as predictor variables; Table S4: Results of Welch’s two sample *t*-test to compare mean of testing and training RMSE of the two models used to predict MEP_amp_; Table S5: GLM for predicting CV of MEP_amp_ using CV of the other IOC parameters as predictor variables; Table S6: GLM for predicting CV of MEP_amp_ using CV of MT, PS and MEP_max_; Table S7: Results of Welch’s two sample *t*-test to compare mean of testing and training RMSE of the two models used to predict CV of MEP_amp_.
