# Supplementary material for: Inter-Individual Variability in Motor Output Is Driven by Recruitment Gain in the Corticospinal Tract Rather Than Motor Threshold
Source: Brain Sci. 2022 Oct 17;12(10):1401. doi: 10.3390/brainsci12101401 (PMC9599681; doi:10.3390/brainsci12101401)
Supplement: Supplementary file 1 [file brainsci-12-01401-s001.zip › brainsci-1937772-supplementary.pdf]

Supplementary Document

1. *Comparing IOC parameters between ADM and APB groups*

From 84 subjects, after removing IOCs that did not saturate (n=6) we compared the IOC parameters between the two muscle groups ADM (n= 46) and APB (n = 32). We performed Welch's t-test which accounts for the unequal sample sizes between the two groups.

	**MT**	**PS**	**MEP_{amp}**	**MEP_{max}**	**S50**
Hypothesis	0	0	0	0	0
p-value	0.9541	0.0769	0.0736	0.3611	0.4546
C.I.	-4.4502<br>4.7155	-0.4212<br>0.0227	-1.1655<br>0.0555	-1.2037<br>0.4461	-3.0400<br>6.7203
t-stat	0.0577	-1.8267	-1.8362	-0.9213	0.7518
df	71.8001	32.7220	40.9162	52.4615	71.0704

Table S1. Result of Welsch's t-test. Hypothesis = 1 if there is difference between means of the same parameters between different muscle groups (ADM vs. APB). MT = Motor Threshold, PS = Peak Slope, MEP_{amp} = MEP amplitude at 120% MT, MEP_{max} = maximum MEP amplitude of subject, S50 = intensity at which half of MEP_{max} is reached, C.I. = Confidence Interval, df = degrees of freedom.

We did not find any significant difference in the mean of any IOC parameter between ADM and APB muscle groups.

2. *Checking for normal distribution of data*

To test if the IOC parameters are normally distributed, we performed the Shapiro-Wilk test.

	**MT**	**PS**	**120% RMT MEP**	**MEP max**	**S50**
Test Statistic	0.9934	0.8787	0.8894	0.9655	0.9873
p value	0.9676	<0.0001	<0.0001	0.0388	0.6610
Normality	Normal	Not Normal	Not Normal	Not Normal	Normal

Table S2. Results of the Shapiro-Wilk test. Only MT and PS showed normal distribution. MT = Motor Threshold, PS = Peak Slope, MEP_{amp} = MEP amplitude at 120% MT, MEP_{max} = maximum MEP amplitude of subject, S50 = intensity at which half of MEP_{max} is reached

3. *Correlation matrix with regression lines*

Figure S1. Correlation matrix with regression line. Each box depicts the correlation between two IOC parameters along with regression lines. The values of the parameters have been log transformed to obtain normal distribution as shown in the bar plots. Pearson's correlation coefficient is displayed for each pair of parameters. The values in red show significant correlation. ($p < 0.05$)

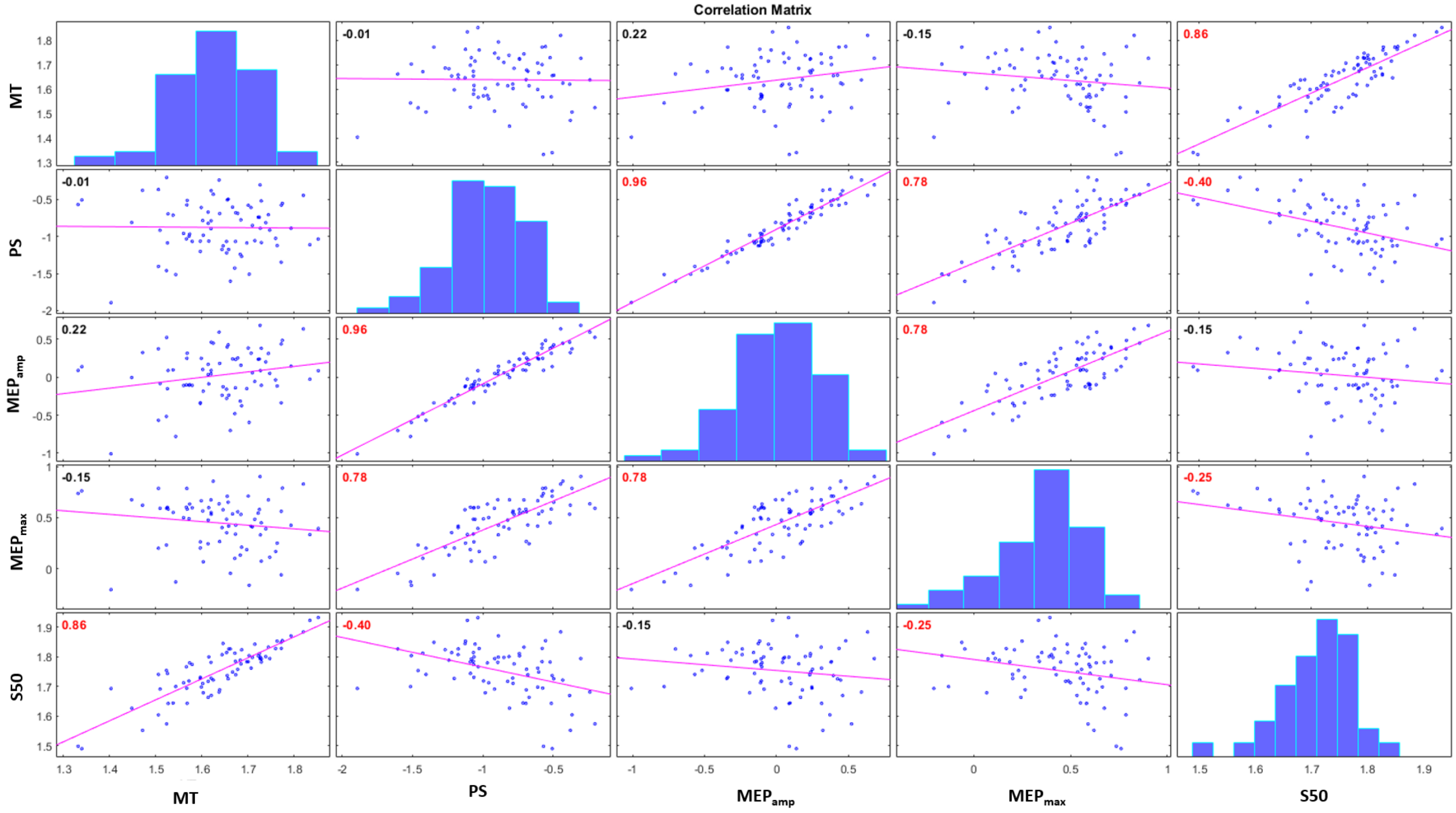

Correlation Matrix
MT
PS
MEP_{amp}
MEP_{max}
S50
-0.01
0.22
-0.15
0.86
0.96
0.78
-0.40
-0.25

4. *Model comparisons for predicting MEP_{amp}*

A GLM was built using MEP_{amp} as dependent variable and z-score normalized MT, PS, MEPmax and S50 as predictor variables. The model had the following properties: residual standard error (SE) = 0.2312 on 70 degrees of freedom, r2 = 0.9471, adjusted r2 = 0.944, F(4,70) =313, p < 0.0001*** (Table S3).

	**Coefficient (β)**	***Standard Error* (SE)**	**t-Statistic**	**p-value**	**VIF**
Intercept	1.3733	0.0267	51.444	<0.0001***	
MT	0.2794	0.0800	3.492	0.0008	8.8651
PS	0.7906	0.0591	13.388	<0.0001***	4.8289
MEP_{max}	0.2149	0.0429	4.999	0.0612	2.5588
S50	0.0489	0.0884	0.554	0.5815	10.8144

Table S3. GLM of MEP_{amp} with MT, PS, MEP_{max} and S50 as predictor variables.

In this table the VIFs of S50 and MT were 10.814 and 8.865 indicating very high collinearity, making the coefficients non-interpretable in the GLM. Hence, another GLM was generated after removing S50 which had the highest VIF (table provided in main manuscript).

The GLM using all the IOC parameters as predictors for MEP_{amp} had similar performance to the GLM which used only the predictors PS, MT and MEP_{max} when validated using 5-fold cross validation. There was no significant difference of variance between the two groups (F(4, 4) = 2.323, p = 0.4344). Hence, a two sample Student's t-test was performed (t(8) = 0.34712, p = 0.7375). No significant difference was found between the mean of the two groups.

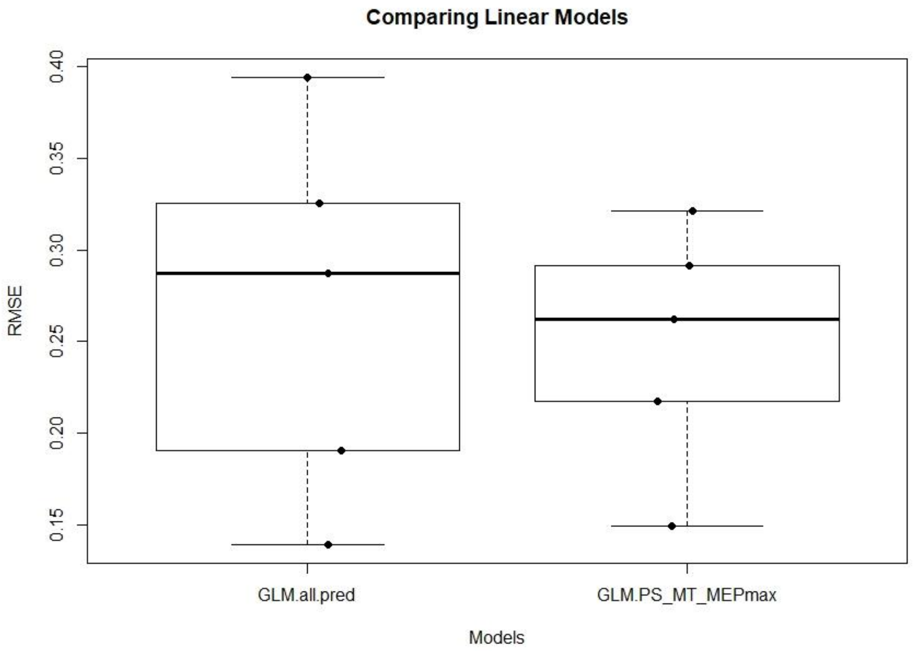

Figure S2. Performance of two different GLMs predicting MEP_{amp}. Dots scattered within box plots represent root mean square error (RMSE) for each fold of 5-fold CV of the model. The solid line represents median RMSE while the whiskers represent maximum and minimum value. GLM (all predictors): mean RMSE ±SD = 0.2674 ±0.1027; GLM (CV of PS and CV of MEP_{max}): mean RMSE ±SD = 0.2483 ±0.0674.

The difference in prediction accuracy did not differ significantly upon removing S50. However, the standard deviation decreased substantially in the model with fewer predictors.

To check for overfitting, the mean RMSE of the training and testing folds of 5- fold cross validation was compared for both the models. To remove bias introduced when randomly shuffling the folds during cross validation, we ran the models for 1000 times and then compared the total mean of RMSE values. Unequal variance was observed in between the training vs testing groups in both models predicting MEP_{amp}:

GLM testing vs. training: $F(4999, 4999) = 17.918$, $p < 0.0001$; RF model testing vs. training: $F(4999, 4999) = 86.12$, $p < 0.0001$.

Hence, Welch's two sample t-test was performed to compare the RMSE means.

	**t statistic**	**df**	**p value**	**Mean (± SD) of Testing Dataset**	**Mean (± SD) of Training Dataset**
GLM Model	29.475	5555.3	<0.0001	0.2453 (0.0583)	0.2203 (0.0138)
RF model	95.609	5115.1	<0.0001	0.3499 (0.1338)	0.1680 (0.0144)

Table S4. Results of Welch's two sample t-test to compare mean of testing and training RMSE of the two models used to predict MEP_{amp}.

Both models had significant difference between testing and training RMSE. However, the RF model had an almost 3-fold lower training RMSE as compared to its testing RMSE and also a 10-fold lower standard deviation for the same. This strongly hints that the RF model might have overfitted to the training data. The GLM training vs testing RMSE means had comparable values.

5. *Model comparisons for predicting CV of* MEP_{amp}

To measure the variability within the entire dataset, the coefficients of variation (CV) of all the IOC parameters were calculated. The CV values are displayed in Figure 3.

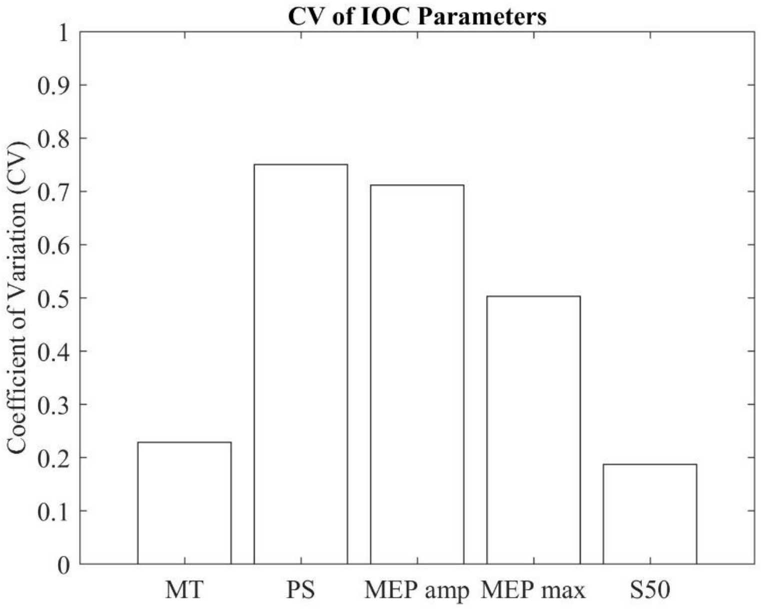

Figure S3. Coefficients of variation (CV) for the IOC parameters. MT CV = 0.2333, PS CV = 0.7511, MEP_{amp} CV = 0.6526, MEP_{max} CV = 0.5105, S50 CV = 0.1871.

A distribution of CV values was obtained using bootstrapping without replacement for all the IOC parameters within our dataset. A GLM was built using these CV values of the IOC parameters to predict CV of MEP_{amp}. We then determined the GLM parameters from 10-fold cross validation model training using bootstrapped data. The model had the following properties: residual SE = 0.0248 on 995 degrees of freedom, r2 = 0.6732, adjusted r2 = 0.6719, F(4,995) = 512.4, p< 0.0001*** (Table S5).

	**Coefficient(β)**	***Standard Error* (SE)**	**t-Stat**	**p-value**	**VIF**
Intercept	0.0116	0.0224	0.520	0.603	
CV of MT	0.9584	0.1083	8.848	<0.0001***	3.6386
CV of PS	0.4907	0.0180	27.217	<0.0001***	1.1313

CV of MEP_{max}	0.6635	0.0314	21.155	<0.0001***	1.2082
CV of S50	-1.1859	0.1239	-9.569	<0.0001***	3.6727

Table S5. GLM for predicting CV of MEP_{amp} using CV of the other IOC parameters as predictor variables.

The results show that CV of S50 and MT both have VIF > 2.5, indicating collinearity. We removed the predictor with the largest VIF (S50, VIF = 3.6727) and ran the model again. It had the following properties: residual SE = 0.0259 on 996 degrees of freedom, r2 = 0.6431, adjusted r2 = 0.642, F(3,996) = 598.3, p < 0.0001*** (Table 6).

	**Coefficient(β)**	***Standard Error* (SE)**	**t-Stat**	**p-value**	**VIF**
Intercept	-0.0423	0.0226	-1.872	0.0616	
CV of MT	0.0779	0.0597	1.306	0.192	1.0127
CV of PS	0.4808	0.0188	25.577	<0.0001***	1.1276
CV of MEP_{max}	0.7460	0.0315	23.688	<0.0001***	1.1168

Table S6. GLM for predicting CV of MEP_{amp} using CV of MT, PS and MEP_{max}.

The GLM using all the IOC parameters as predictors for MEP_{amp} had similar performance to the GLM which used only the predictors PS, MT and MEP_{max} when validated using 5-fold cross validation. There was no significant difference of variance between the two groups (F

(9, 9) = 2.323, p = 0.2671). Hence, a two sample Student's t-test was performed ($t(18) = -1.1282$, $p = 0.274$). No significant difference was found between the mean of the two groups.

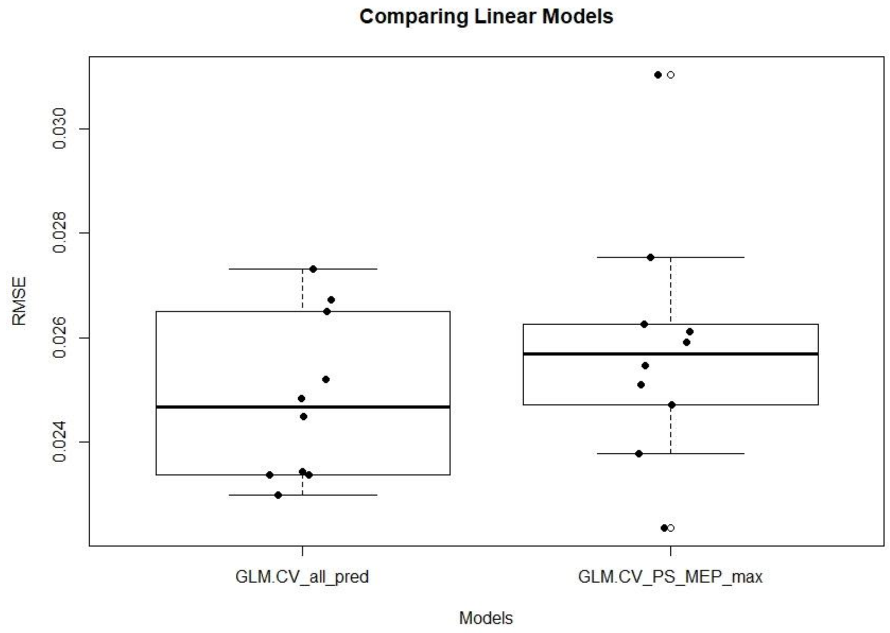

Figure S4. Performance of two different GLMs predicting CV of MEP_{amp}. Dots scattered within box plots represent root mean square error (RMSE) for each fold of 10-fold CV of the model. The solid line represents median RMSE while the whiskers represent maximum and minimum value. GLM (all predictors): mean RMSE ±SD = 0.0248 ±0.0016; GLM (CV of PS and CV of MEP_{max}): mean RMSE ±SD = 0.0258 ±0.0023.

The difference in prediction accuracy did not differ significantly upon removing CV of S50 and CV of MT. The regression lines between CV of MEP_{amp} and its two final predictors were plotted in the correlation matrix below.

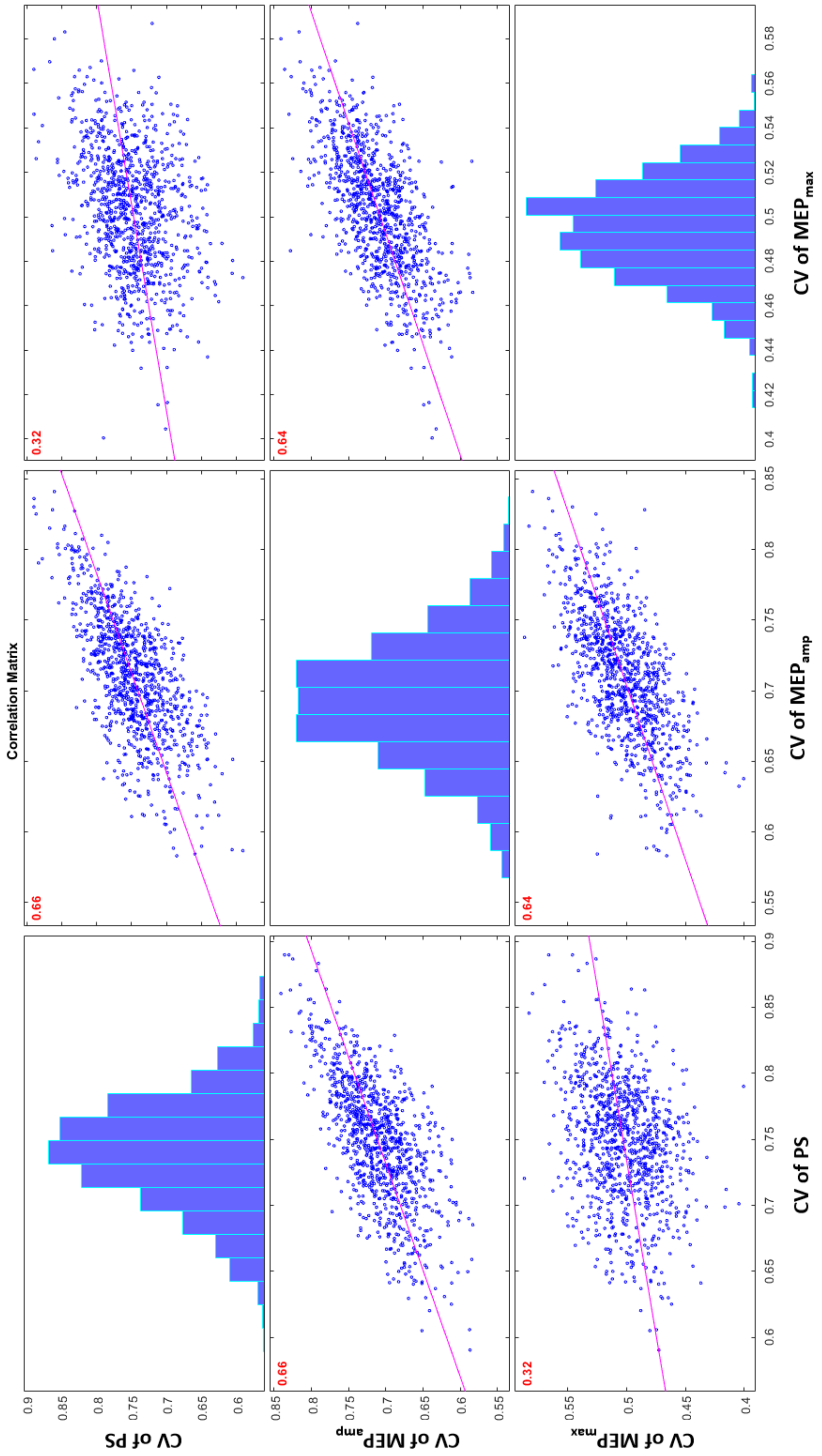

Figure S5. Regression lines between the final two predictors of MEP_{amp} CV. The red-coloured values represent significant Pearson's correlation coefficient ($p < 0.05$). The values of the coefficients are: CV of PS vs. CV of MEP_{max} = 0.32, CV of PS vs. CV of MEP_{amp} = 0.66, CV of MEP_{max} vs. CV of MEP_{amp} = 0.64.

To check for overfitting, the mean RMSE of the training and testing folds of 10- fold cross validation was compared for both the models. To remove bias introduced when randomly

shuffling the folds during cross validation, we ran the models for 1000 times and then compared the total mean of RMSE values. Unequal variance was observed in between the training vs testing groups in both models predicting the CV of MEP_{amp}:

GLM testing vs. training: $F(9999, 9999) = 80.149$, $p < 0.0001$; RF model testing vs. training: $F(9999, 9999) = 268.4$, $p < 0.0001$. Hence, Welch's t-test was used to compared mean RMSE.

	**t statistic**	**df**	**p value**	**Mean (± SD) of Testing Dataset**	**Mean (± SD) of Training Dataset**
GLM Model	3.0692	10248	0.0021	0.0248 (0.0583)	0.0247 (0.0138)
RF model	749.11	10074	<0.0001	0.0262 (0.1338)	0.0118 (0.0144)

Table S7. Results of Welch's two sample t-test to compare mean of testing and training RMSE of the two models used to predict CV of MEP_{amp}.

Both models had significant difference between testing and training RMSE. However, the RF model had an almost 2-fold lower training RMSE as compared to its testing RMSE and also about a 10-fold lower standard deviation for the same. This strongly hints that the RF model might have overfitted to the training data. The GLM training vs testing RMSE means had comparable values.

6. *Relationship between the mean and CV of all IOC parameters in bootstrapped data*

Figure S6. Regression lines between all IOC parameters along with their Pearson's correlation coefficients. (* = significance of <0.5, ** = significance of <0.05, ***= significance of < 0.001)

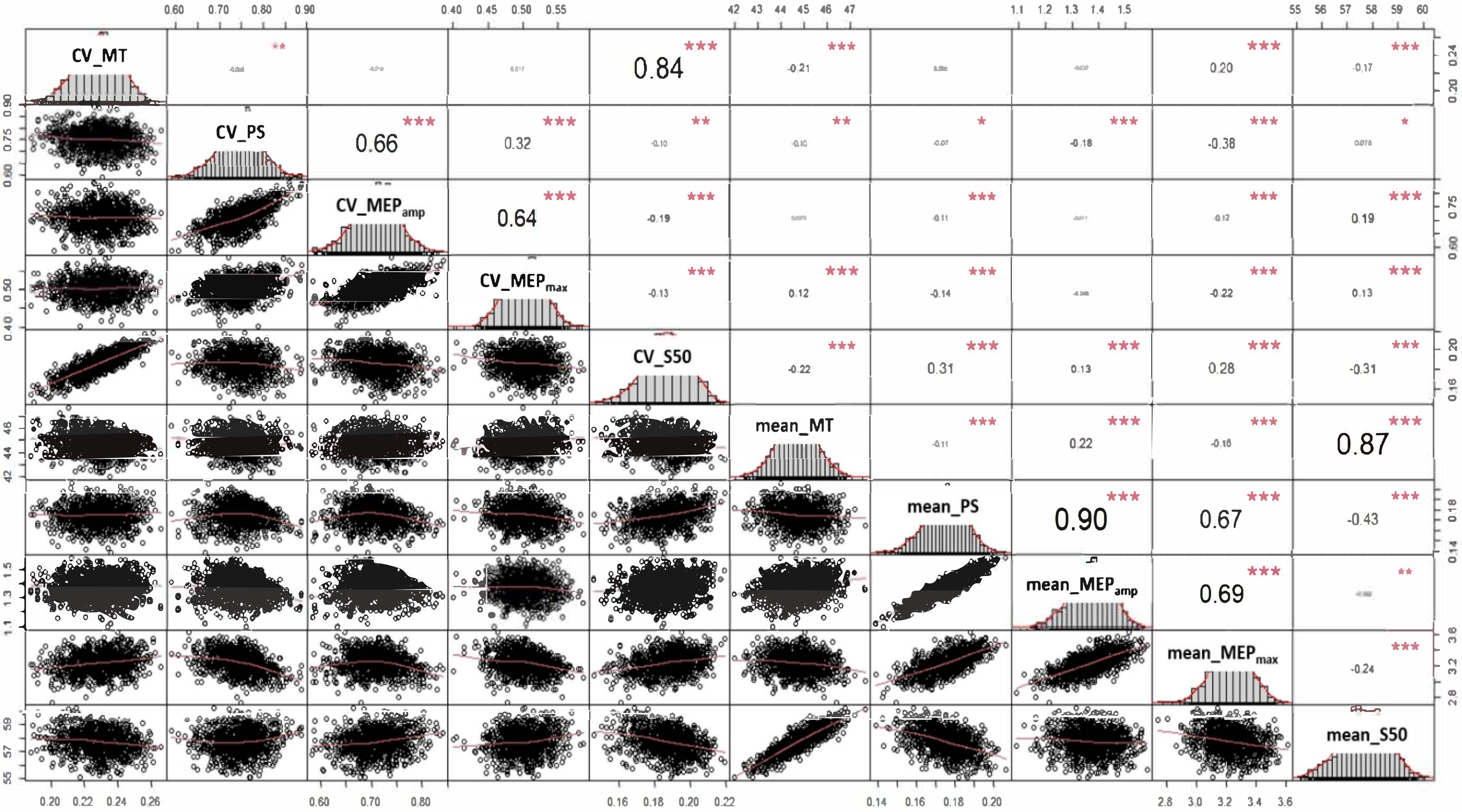

CV_MT
CV_PS
CV_MEP_{amp}
CV_MEP_{max}
CV_S50
mean_MT
mean_PS
$mean\_MEP_{amp}$
$mean\_MEP_{max}$
mean_S50
0.84
-0.21
0.20
-0.17
0.66
0.32
-0.18
-0.38
0.64
-0.19
0.19
-0.13
0.12
-0.14
-0.22
0.13
-0.22
0.31
0.13
0.28
-0.31
0.22
0.87
0.90
0.67
-0.43
0.69
-0.24
